# SNRNP200 Mutations Cause Autosomal Dominant Retinitis Pigmentosa

**DOI:** 10.3389/fmed.2020.588991

**Published:** 2021-01-21

**Authors:** Tao Zhang, Jingshan Bai, Xinyi Zhang, Xiaowei Zheng, Nan Lu, Zhongyin Liang, Ling Lin, Yongsong Chen

**Affiliations:** ^1^Department of Ophthalmology, The First Affiliated Hospital of Shantou University Medical College, Shantou, China; ^2^The Clinical Research Center of the First Affiliated Hospital of Shantou University Medical College, Shantou, China; ^3^Department of Ophthalmology, The Second People's Hospital of Dongying, Dongying, China; ^4^Department of Ophthalmology, The Dawang Hospital of Guangrao of Dongying, Dongying, China; ^5^Department of Cardiology, The First Affiliated Hospital of Shantou University Medical College, Shantou, China; ^6^Department of Bioinformatics, Berry Genomics Co., Ltd., Beijing, China; ^7^Department of Rheumatology, The First Affiliated Hospital of Shantou University Medical College, Shantou, China; ^8^Department of Endocrinology, The First Affiliated Hospital of Shantou University Medical College, Shantou, China

**Keywords:** retinitis pigmentosa, SNRNP200, whole exome sequencing, mutation, morpholino oligonucleotide

## Abstract

The small nuclear ribonucleoprotein 200 kDa (SNRNP200) gene plays a key role in the maturation of pre-message RNA (pre-mRNA) splicing with the indication for the etiology of retinitis pigmentosa (RP). Gene recognition can facilitate the diagnosis of these patients for better clinical management, treatment and counseling. This study aimed to outline the causative mutation in a Chinese family and the pathogenic mechanism of this SNRNP200 mutation in RP. Eighteen individuals from the affected family underwent a complete ophthalmic examination. Whole exome sequencing (WES) was conducted to identify the pathogenic variant in the proband, which was then confirmed by Sanger sequencing. Expression of the SNRNP200 transcript in zebrafish was identified via whole mount *in situ* hybridization. Morpholino oligonucleotide (MO) and SNRNP200 wild and mutant mRNA were injected into zebrafish embryos followed by analyses of the systemic changes and retinal phenotypes using immunofluorescence. Heterozygous SNRNP200^c.C6088T^ (p.Arg2030Cys) mutation was ascertained in two members of this family: the proband and his father (II-2). Overexpression of SNRNP200^Arg2030Cys^, but not SNRNP200^WT^ caused systemic deformities in the wild-type zebrafish embryos with the retina primarily injured, and significantly increased death rates in the morphant embryos, in which the orthologous zebrafish SNRNP200 gene was blocked. In conclusion, this study reports a novel heterozygous SNRNP200^c.C6088T^ mutation, which is evidenced to cause RP via a dominant-negative effect.

## Introduction

Retinitis pigmentosa (RP) is reported as the most regular form of inherited degenerative retinal dystrophy, with a prevalence ranging between 1/3,500 to 1/5,000 among different countries worldwide (1, 2). Nyctalopia is one of the earliest and most common symptoms of RP, followed by subsequent constricted visual fields (VFs), and eventual loss of central vision caused by the degeneration of photoreceptor and retinal pigment epithelium (RPE) (3, 4). The fundus in RP is characterized by peripheral bone-spicule pigmentary deposits, attenuation of the artery, and waxy pallor of the optic nerve head. Outer nuclear layer attenuation and the loss of outer/inner segments of RPE in the macular area of the retina are the typical characteristics. The inheritance of RP could be in three modes, autosomal dominant RP (adRP), autosomal recessive RP, and X-linked RP. Thus far, 307 genes and gene loci have been shown to be involved in retina degeneration [Retnet database: https://sph.uth.edu/retnet/; reviewed in Daiger et al. (5)]. The majority of these genes are specifically expressed in the retina. Interestingly, 6 of 22 adRP-related genes code for universally expressed pre-mRNA splicing proteins that are essential splicing factors, called the small nuclear ribonucleoprotein particles (snRNPs). These genes include PRPF6 (MIM 613979) (6), PRPF31(MIM 606419) (7), PRPF8 (MIM 607300) (8), PRPF3 (MIM 607301) (9), PIM1-associated protein [RP9 (MIM 607331)] (10), and small nuclear ribonucleoprotein 200 kDa (SNRNP200) (11, 12).

For most eukaryotic genes, the primarily transcribed RNA from the gene's DNA must be edited through a process called splicing before it becomes mature, and only then can it guide the synthesis of proteins. During the process of primary RNA editing, the sequence of the introns will be removed and the sequence of the exons will be connected together, through the actions of the spliceosome, primarily comprising U1, U2, U4/U6, and U5 snRNPs. The complex of U4/U6–U5 tri-snRNP is essential for installing and the catalytic process of the spliceosome structural rearrangements. Thus, any defect in the complex could possibly contribute to the pathogenesis of RP (13).

SNRNP200 encodes hBrr2, which is one of the U5 snRNP-specific proteins (NP_054733.2) (14), containing 2,136 amino acids (15) and catalyzing the U4/U6 unwinding (16). It has been reported in the literature that the mutation of SNRNP200 can compromise the U4/U6 unwinding (11) and when blocked, could cause the demorphogenesis of rod photoreceptors in a zebrafish model (17). However, the exact pathogenic mechanism of the SNRNP200 mutations has never been demonstrated.

Herein, we report one naturally occurring heterozygous mutation in SNRNP200, c.C6088T (p.Arg2030Cys), which associates with adRP in a Chinese family and investigate the pathogenic mechanism of this SNRNP200 mutation.

## Materials and Methods

### Study Subjects and Clinical Examinations

The methods in this study were approved by the Dawang hospital of Guangrao of Dongying Institutional Review Board and complied with the local laws (Certificate NO. DWZWSY2019-05). The study was performed following the Declaration of Helsinki and the standards of the Institutional Animal Care and Use Committee, Institutional Animal Ethics Committee. Written informed consent was obtained from all the study participants.

**Figure 1:**
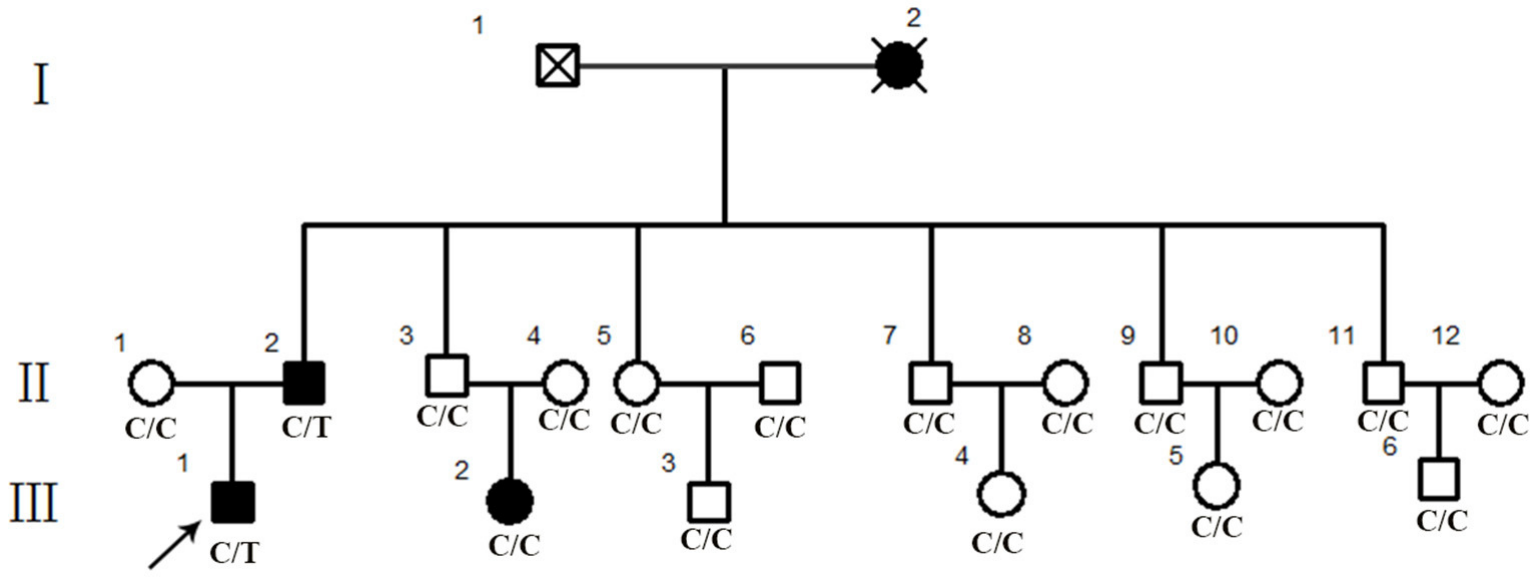
Pedigree of a Chinese retinitis pigmentosa families. Pedigree representing three consecutive generations of members in a Chinese family with typical phenotype of adRP. Filled symbols represent affected individuals; open symbols represent unaffected family members.

A Chinese family (Figure 1) diagnosed with RP consisting of three affected patients and 15 unaffected family members was recruited from Dawang hospital of Guangrao of Dongying. Venous blood samples were collected for whole exome sequencing (WES) from family members and 400 unrelated Chinese individuals without ocular diseases serving as controls.

Very detailed ophthalmic examinations, including routine slit-lamp examination, visual acuity, intraocular pressure, fundus photography, visual field, and optical coherence tomography (OCT) in the macula, were conducted.

### Animals

Zebrafish (Danio rerio) were maintained and reared under standard conditions-28.5 °C in a 14 h light and 10 h darkness rotation in the Model Animal Research Center. All the embryos used in experimenting were obtained by natural mating in the morning. Animal experiments were approved by the Dawang hospital of Guangrao of Dongying Animal Care commission and the local ethical review board and conformed to the Guideline for the Care and Use of laboratory animals of the National Institutes of Health.

### Genetic Analysis

#### Whole Exome Sequencing and Sanger Sequencing

Genomic DNA was extracted from peripheral blood cells as per the protocols of the Lab-Aid DNA kit (Tiangen Biotech Co., Beijing, China). The target exomes capture was performed using the Agilent SureSelect Human All Exon V5 Kit (Agilent Technologies, Santa Clara, CA), and sequencing was performed on an Illumina Hiseq2500 (Illumina, San Diego, CA, USA) following the manufacturer's protocol. The results of the selected pathogenic variants were confirmed by bidirectional Sanger sequencing.

#### Expression Analyses of SNRNP200

Quantitative real-time PCR (Q-PCR) and reverse-transcriptase polymerase chain reaction (RT-PCR) were conducted to investigate the expression of SNRNP200 in multiple tissues of the human cadaver eye including the cornea, iris, lens, vitreous body, retina, optic nerve and others; a total RNA extraction from these samples was performed using TRIZOL reagent. cDNA synthesis was performed using a TOYOBO RT-PCR kit (FSQ-301, TOYOBO, Japan) per the manufacturer's protocol. The expression profile of SNRNP200 was analyzed using the following primers: forward (5′-AGCTCTTTGCTGCCTGTGTC-3′), reverse (3′-TACCAAGCACCTAGCCAATG-5′); GAPDH forward (5′-TCATTGACCTCAACTACATGG−3′), reverse (3′-TCGCTCCTGGAAGATGGTG-5′).

#### Functional Analyses of SNRNP200 Mutation in Zebrafish

##### Microinjections of Zebrafish

Capped and tailed mRNAs of SNRNP200 ^WT^ (hSnrnp200 ^WT^) and SNRNP200 ^c.C6088T^ (hSnrnp200 Arg2030Cys) were achieved from the circular plasmids cut by the endonuclease XmaI AcpxT7- SNRNP200^WT^ and AcpxT7- SNRNP200^c.C6088T^ with the mMESSAGE MACHINE T7 Ultra Kit (Ambion, Austin, TX, USA). RNeasy Kit (Qiagen, Hilden, Germany) was employed to erase extra nucleotides, especially DNA aiming at purifying the synthesized mRNAs.

#### Blocking MOs for Zebrafish

Snrnp200-MO (5′-TCAACATCAAGACAACTCACATCCT-3′) and Control-MO (5′-CCTCTTACCTCAGTTACAATTTATA-3′) were designed as previously described and purchased from Gene Tools, LLC (Philomath, Oregon, USA).

Embryos at 1 cell-stage were chosen for microinjections in the morning. For mRNA microinjection, each embryo was injected with 1 nL solution containing 200 pg SNRNP200^WT^ or SNRNP200^c.C6088T^ mRNA. For co-injections of MO and mRNA, zebrafish embryos were separated into four groups and were injected with 4 ng Control-MO, 4 ng Snrnp200-MO, 4 ng Snrnp200-MO plus 200 pg SNRNP200^WT^ mRNA, and 4 ng Snrnp200-MO plus 200 pg SNRNP200^c.C6088T^ mRNA, respectively. During the entire experiment, the embryos that died within 24 h postoperatively were eliminated, as it was likely that they died from indefinite causes. The percentage of deformation and mortality of zebrafish embryos was calculated from 2 to 3 dpf.

#### Expression Analysis of the Immunofluorescence of Zebrafish

At 3 dpf, zebrafish were randomly selected from the experiment groups. Immunostaining was performed to evaluate their ocular morphology and rod photoreceptors. The zebrafish embryo was fixed in 4% paraformaldehyde (PFA) overnight at 4°C. On the second day, the fixed ones were washed three times with phosphate-buffered saline (PBS) containing 0.01% Tween 20 (PBST) and dehydrated in 30% sucrose overnight at 4°C. Subsequently, they were embedded in an optimal cutting temperature solution (Invitrogen, Carlsbad, CA, USA) at −40°C for 2 h before being cut at −10°C using the Leica CM1860 cryostat (Leica Microsystems, Nussloch Germany). For immunostaining, the slides were dehydrated for 30 min at 37°C, rehydrated with PBST for 5 min, and blocked with PBS supplemented with 10% normal goat serum and 2% bovine serum albumin for 1 h at room temperature. The slides were then incubated with antizebrafish rhodopsin overnight at 4°C. A confocal laser scanning microscope (Nikon, Tokyo, Japan) was applied for image collection.

## Results

### Clinical Evaluations of the Patients With SNRNP200 Variant

**Figure 2:**
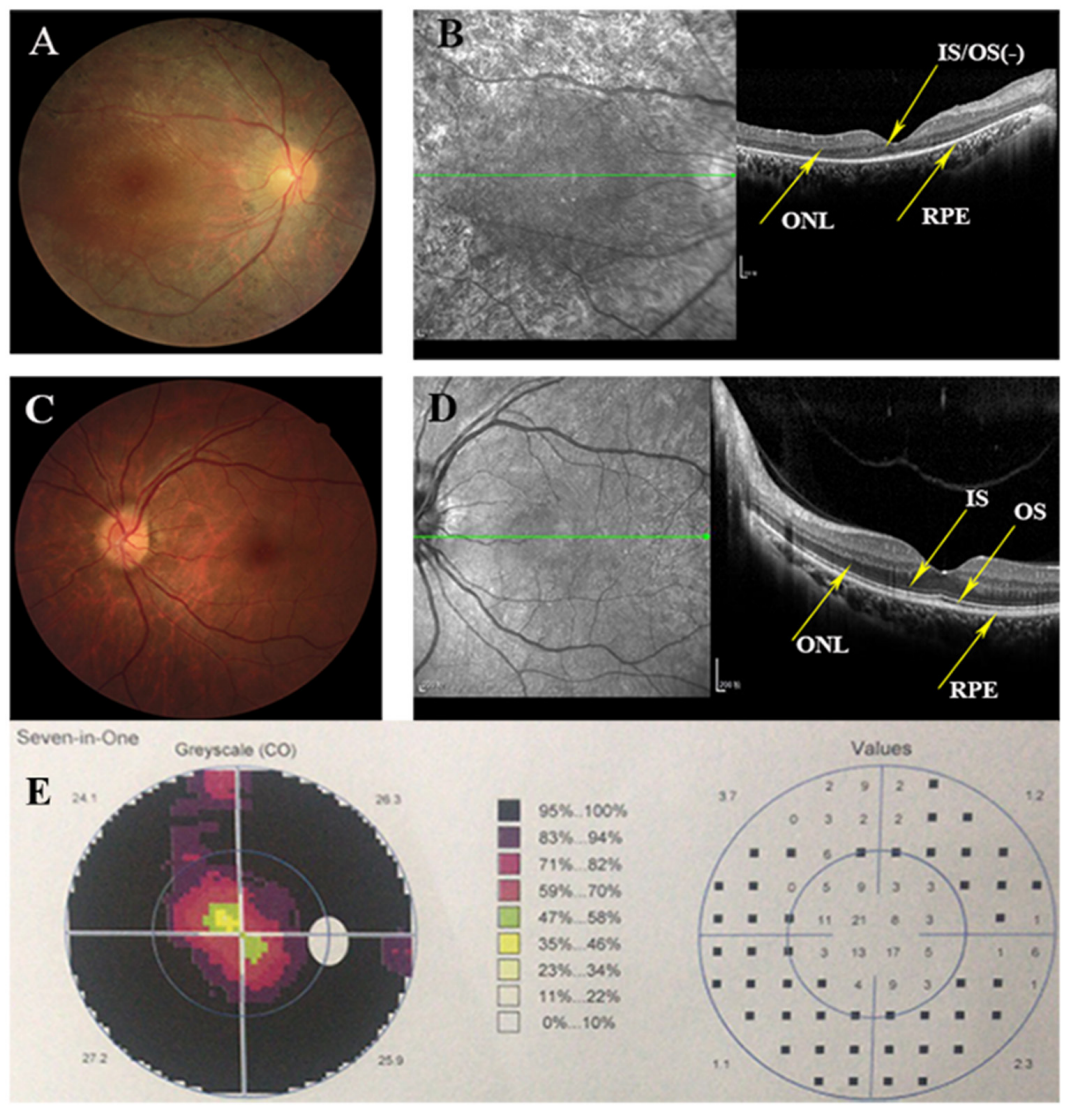
Clinical information of this adRP family. **(A)** Right eye fundus of the proband (III:1) presents characteristic RP degeneration, including a waxy pallor of optic disc, attenuated artery and vein and peripheral pigment deposit. **(B)** OCT scan reveals macular degeneration showing reduced thickness of outer nuclear layer (ONL) and RPE at the macular area with partial loss of outer/inner segments (OS/IS). **(C,D)** Fundus picture and Optical coherence tomography (OCT) scans of the left eye of the unaffected member (III:3) showing as normal controls. **(E)** Visual fields of the right eye of the proband indicates the tubular visual field.

Eighteen individuals from a family were recruited, following the adRP inheritance (Figure 1). The proband III:1 was first referred to an ophthalmic clinic at the age of 7 years for his night vision disorder. Afterward, his VFs and vision deteriorated further with time. At his latest visit at the age of 27 years, he demonstrated a typical RP fundus, including waxy pallor of the optic disk, attenuated artery and vein, and peripheral pigment deposit (Figures 2A vs. C). The reduced thickness of the outer nuclear layer and RPE at the macular area with partial loss of outer/inner segments (OS/IS) were demonstrated by OCT (Figures 2B vs. D), all of which corresponded to his poor visual acuity (Table 1). Patients II:2 and III:2 developed nyctalopia at 5 and 9 years of age, respectively. During the last examination at ages of 48 and 32 years, respectively, their phenotypes were similar to those of the proband, including inferior night vision, inferior central visual acuity, significantly reduced VFs (Figure 2E), and typical RP fundus appearances. The details of the clinical information of this family are presented in Table 1.

**Table 1:** The character of the affected patients.

**P-ID**	**Genotype**	**OA (year)**	**Age (year) /sex**	**BCVA (logMAR)**		**Refractive error**		**Fundus appearance**							**VF**		
				**OD**	**OS**	**OD**	**OS**	**OD MD**	**OD**	**AA**	**MD**	**PD**	**OS MD**	**AA**	**PD**	**OD**	**OS**
II-2	c.6088C>T	5	48/M	FC	HM	−3.0DS/−3.0DC × 180°	−2.0DS/-1.0DC × 15°	SEV	Y	Y	SEV	Y	Y	Y	Y	T	T
III-1	c.6088C>T	7	27/M	0.3	0.3	+0.5DS/−2.25DC × 165°	+0.75DS/-3.25DC × 5°	MOD	Y	Y	Y	Y	Y	Y	Y	T	T
III-2	No	9	32/F	0.4	0.3	−2.5DS/−1.0DC × 120°	−1.5DS/-1.0DC × 60°	MOD	Y	Y	Y	Y	Y	Y	Y	T	T

*P-ID, patient ID; OA, onset age; No, no mutation F, female; M, male; BCVA, best corrected visual acuity; logMAR, logarithm of the minimum angle of resolution; HM, hand moving; OD, right eye; OS, left eye; MD, macular degeneration, OD, optic disk, PD, pigment deposits, MOD, moderate; AA, artery attenuation; NOR, normal; SEV, severe*.

### Identification of Putative Variants in SNRNP200

**Figure 3:**
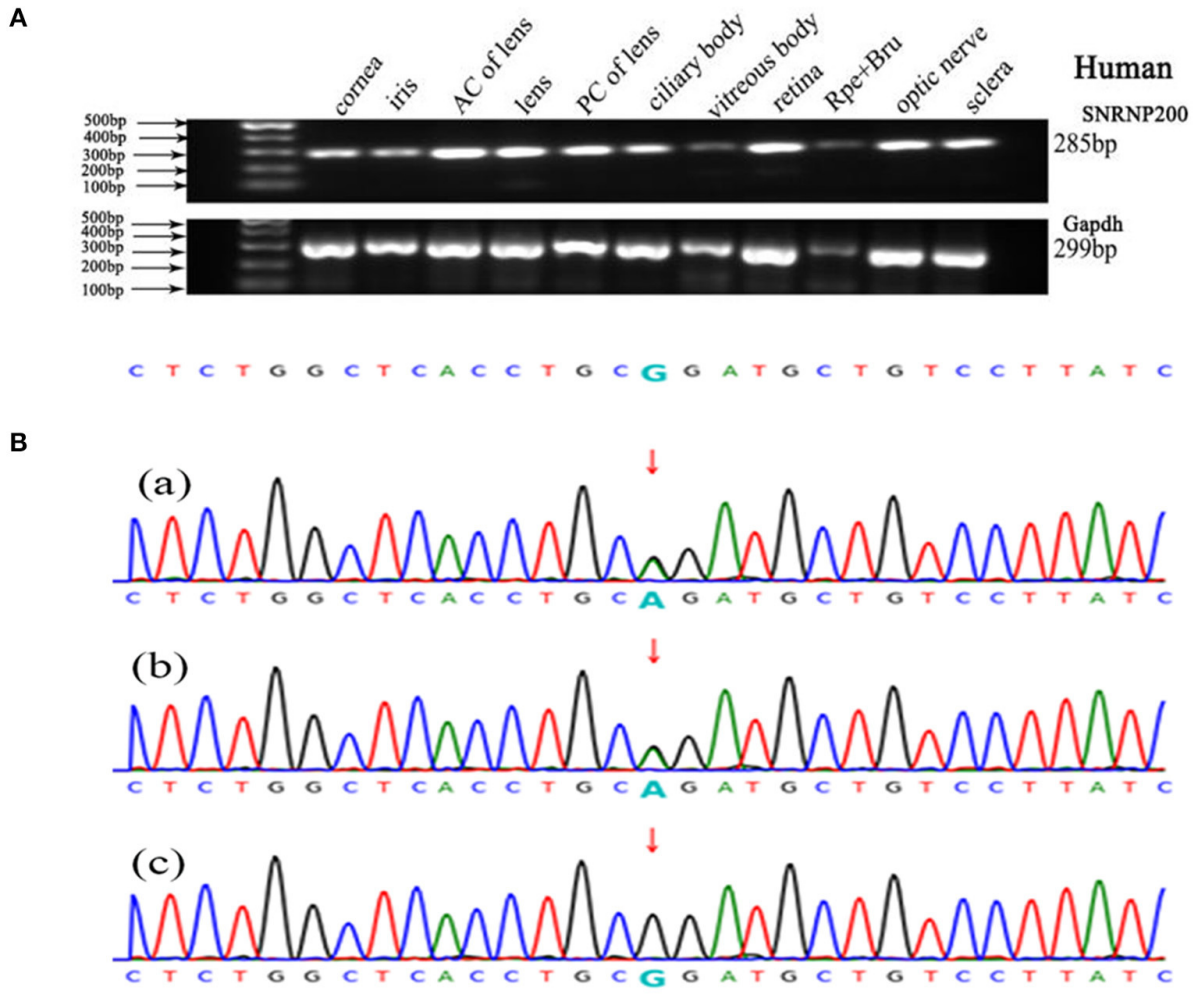
Genetic analyses of variants identified in the SNRNP200 Gene and its expression of SNRNP200 in cadaver eye tissues. **(A)** Expression of SNRNP200 in multiple cadaver eye tissues including cornea, iris, anterior capsule of lens (AC of lens), lens, posterior capsule of lens (PC of lens), ciliary body, vitreous body and neural retina and so on. retinal pigmented epithelium (RPE) is shown. A 285 bp PCR product of the SNRNP200 was detected in all tissues. A 299 bp PCR products of the human Gapdh were selected in parallel as control. **(B)** Sanger sequencing confirmation of the identified SNRNP200 mutations from the whole exome sequencing analysis in the recruited Chinese retinitis pigmentosa families. Sanger sequencing validation of the SNRNP200 mutations (c.6088C>T) identified by the WES analysis in this family.

We performed WES using the genomic DNA of proband III-1 (Figure 1). A total of 51,767 variants were initially identified in the proband, including 47,875 single nucleotide variations, and 3,892 insert/deletions (Indels). The coverage of the targeted region was 96% at least 20 ×. Finally, only one putative pathogenic variant, heterozygous SNRNP200 (NM_014014.4) c.C6088T, which cosegregated with the disease phenotype in the family was identified (Figures 1, 3B). This variant was not present in all single nucleotide polymorphism databases and 400 unrelated controls as demonstrated by Sanger sequencing.

### Ubiquitous Expression of SNRNP200 in the Human Eye and Zebrafish

**Figure 4:**
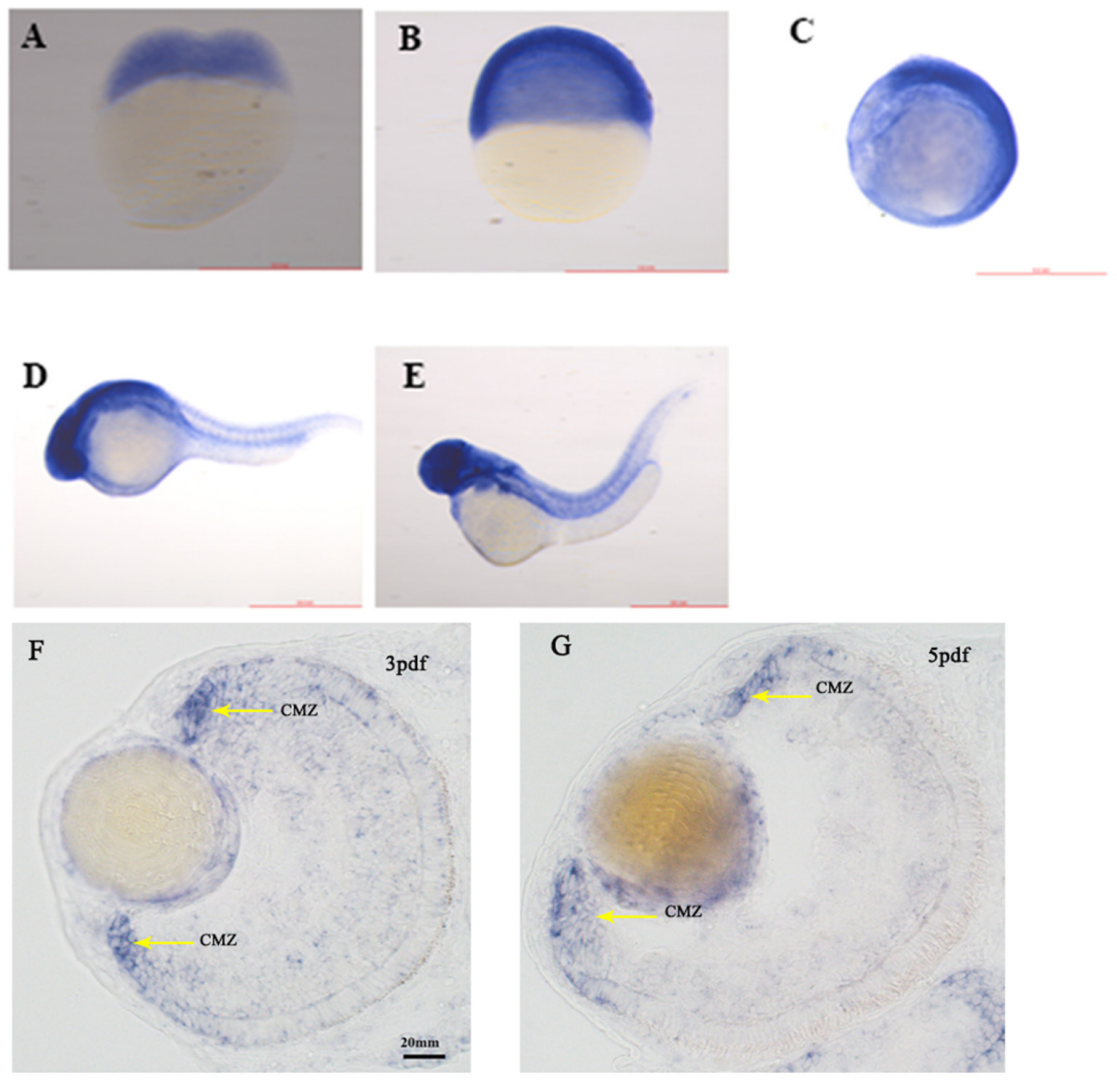
The expression of SNRNP200 in zebrafish. Whole mount *in situ* hybridization revealed the expression of the Snrnp200 gene of zebrafish at the two-cell stage **(A)**, 50%-epiboly **(B)**, 8-somite stage **(C)**, 1pdf **(D)**, and 2pdf **(E)**. *In situ* hybridization (ISH) of zebrafish retinal cryosections exhibit its expression is enriched at the ciliary marginal zone (CMZ) at 3dpf **(F)** and 5dpf **(G)**.

SNRNP200 has been previously investigated showing a wide expression in multiple tissues of humans and mice by reverse-transcriptase polymerase chain reaction (11). Here, we evaluated the expression pattern of SNRNP200 (NM_014014.4) in a panel of human cadaver eye tissues. As predicted, the SNRNP200 was expressed in many tissues, including the cornea, iris, the anterior capsule of the lens, lens, posterior capsule of the lens, ciliary body, vitreous body, neural retina, and so on (Figure 3A). Moreover, the expression of SNRNP200 in zebrafish was revealed by whole mount *in situ* hybridization among several developmental phases (Figures 4A–E). From 3 dpf, its expression was found to be enriched at the ciliary marginal zone (CMZ) (Figures 4F,G).

### The Detrimental Reaction of SNRNP200 p.Arg2030Cys in Zebrafish

Overexpression of SNRNP200 ^p.Arg2030Cys^ causes deformations in a large fraction of zebrafish.

**Figure 5:**
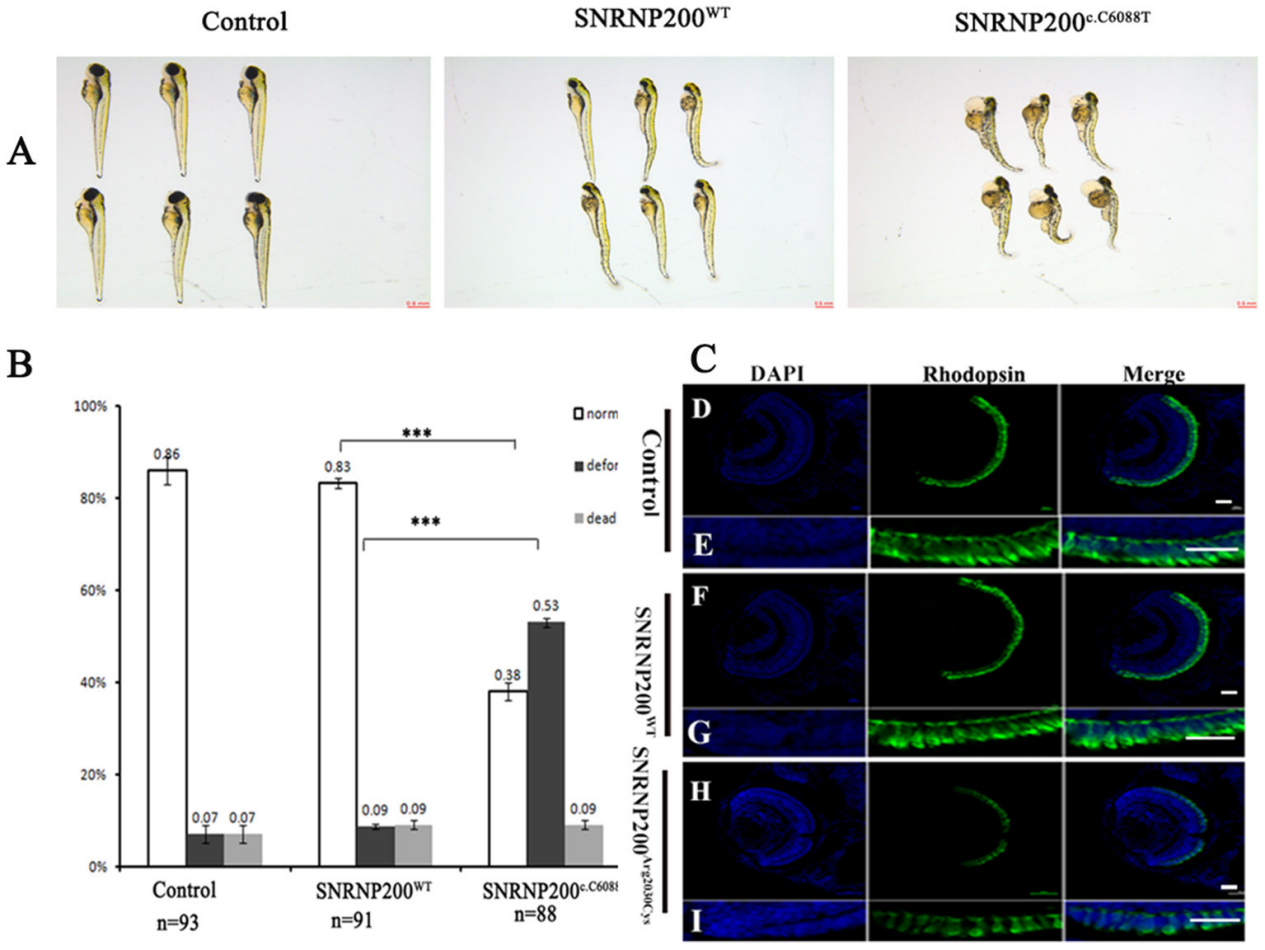
Detrimental effects of SNRNP200 p.Arg2030Cys in zebrafish. **(A)** Morphological changes at 3 dpf after injections of SNRNP200^WT^, SNRNP200 p.Arg2030Cys. There is a significant difference of systemic deformations among groups. **(B)** Statistics of normal, deformed, and dead zebrafish after injection with SNRNP200 p.Arg2030Cys, SNRNP200^WT^ from 2 to 4 dpf. Data are shown as mean + SD from technical triplicates. n: total number of larvae for each group from triple experiment. ***P < 0.001. **(C)** Immunostaining analysis of the zebrafish larvae injected with different mRNAs at 3 dpf for rhodopsin (green). The normal zebrafish revealed abundant expression of rhodopsin in the IS/OS layer **(D–I)** by contrast, the reactivity of rhodopsin was significantly diminished in SNRNP200^c.C6088T^-injected larvae.

SNRNP200^WT^ or SNRNP200^c.C6088T^ mRNA was injected into zebrafish embryos and the one without injection was selected as control. Considering the ubiquitous expression of SNRNP200 with elementary cellular function for immature mRNA splicing, the systemic appearance of the overexpression of SNRNP200 ^p.Arg2030Cys^ and SNRNP200^WT^ were recorded in zebrafish embryos. The group of SNRNP200^c.C6088T^ caused severe defects in the systemic phenotype of zebrafish embryos including the curved body shape, cardiac edema, shortened body trunk, and deformed brain. In comparison with the group of SNRNP200^WT^, there is a significant rise of deformation of larvae in the group of SNRNP200 ^p.Arg2030Cys^ (53% vs. 9%, *p* < 0.0001) (Figures 5A,B). These three groups showed a low percentage of mortality without a significant statistical difference, probably because of the toxicity of exogenous mRNA.

Overexpression of SNRNP200 ^p.Arg2030Cys^ affects the retina of zebrafish.

We studied the effect of overexpression of SNRNP200 ^p.Arg2030Cys^ and SNRNP200^WT^ on the retina. Zebrafish having a normal systemic appearance from both SNRNP200^WT^ and SNRNP200 ^p.Arg2030Cys^ groups at 3 dpf were randomly selected and their retinal phenotype was identified. Certainly, although all the zebrafish showed normal ocular size, the expression of rhodopsin in the group injected with SNRNP200^c.C6088T^ dramatically dropped, as demonstrated by immunofluorescence staining. Conversely, the group of SNRNP200^WT^ revealed a rich expression of SNRNP200^WT^ with a similar amount to the normal group [Figure 5C(D–I)].

Overexpression of SNRNP200 ^p.Arg2030Cys^ significantly increased death rates in zebrafish with SNRNP200 silenced.

**Figure 6:**
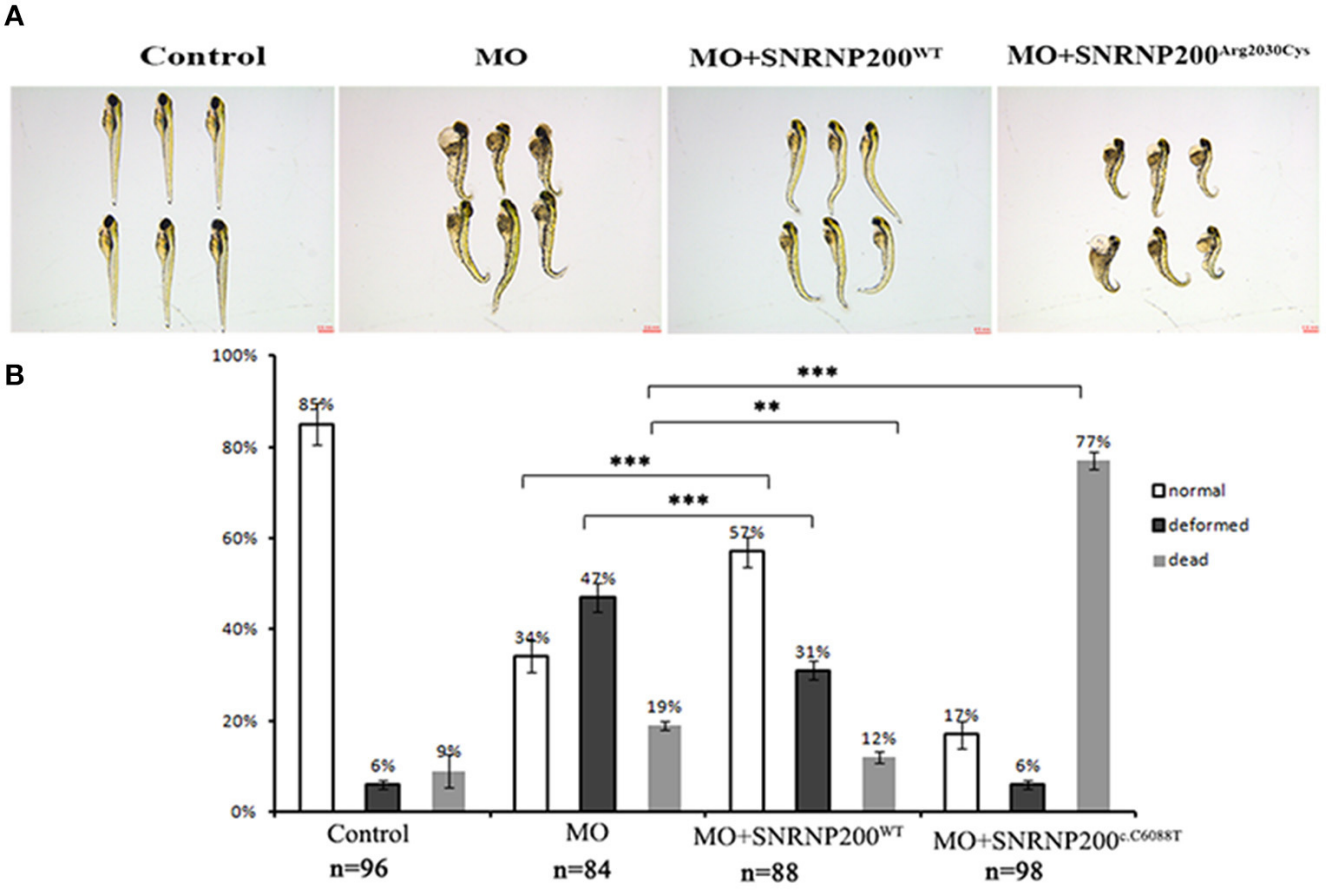
Phenotypic comparison among zebrafish larvae with SNRNP200 silencing, and with overexpression of SNRNP200^WT^, or SNRNP200^c.C6088T^. **(A)** Systemic phenotype of zebrafish larvae injected with 4 ng Control-MO (control); 4 ng SNRNP200-MO (MO); 4 ng SNRNP200-MO +200 pg SNRNP200^WT^ mRNA (MO + SNRNP200^WT^); and 4 ng SNRNP200-MO+200 pg SNRNP200^c.C6088T^ mRNA(MO + SNRNP200^c.C6088T^). **(B)** phenotype distribution in four groups injected variously as shown in **(A)** including normal, deformed and dead ones were calculated from 1 to 3 dpf. The percentage of those are shown in the graph. Comparing with the group injected with SNRNP200-MO, the group of injection of MO + SNRNP200^WT^ particularly reduced the ratio of deformed and dead larvae, whereas injection of MO + SNRNP200^c.C6088T^ significantly raise the rate of death (34 vs. 77%, *P* < 0.001). Data are shown as mean + SD from technical triplicates. n: total number of larvae for each group from triple experiment. **P < 0.01, ***P < 0.001.

The effect of overexpression of SNRNP200^WT^ or SNRNP200^c.C6088T^ in a previously described zebrafish model, in which the orthologous zebrafish SNRNP200 was blocked by the injection of translational blocking morpholino oligos (MOs) into embryos (17) was further investigated. The co-injection of MO with SNRNP200^WT^ or SNRNP200^c.C6088T^ mRNA was implemented and compared with the pure MO-injected and control group. Our results showed that the pure MO-injected group caused a ratio of 47% deformation and 19% death at 3 dpf (Figures 6A,B). As expected, the co-injection of SNRNP200^WT^ definitely could rescue some part of the morphant phenotypes indicated by the reduced ration of deformation and death (31 and 12%, respectively). In contrast to SNRNP200^WT^, the co-injection group of SNRNP200-MO and SNRNP200^c.C6088T^ dramatically increased the frequency of dead morphants to 77% (Figures 6A,B), suggesting that p.Arg2030Cys has a dominant-negative effect, which could hinder the function of zebrafish SNRNP200 despite the loss of function of mutant SNRNP200^c.C6088T^.

## Discussion

The key role of defects in the U4/U6–U5 tri-snRNP complex has been identified by recent genetic and functional research in the process of disease etiology and pathogenic mechanism of RP. SNRNP200, a gene encoding a crucial component of U5 snRNP known as hBrr2 (16), has long been implicated in RP. In this study, we report the correlation of RP with novel naturally occurring heterozygous variants in SNRNP200, which were absent in 400 unrelated controls. Functional analysis of this allele in zebrafish further demonstrates its pathogenic effect having a dominant-negative effect.

In our study, the novel mutation, SNRNP200^c.C6088T^: p.Arg2030Cys was identified in this family by WES, which caused an amino acid change from arginine into cysteine in patients II-2 and III-1. However, the same mutation of this gene was not detected in patient III-2 who had the same clinical signs of RP. Therefore, we infer that her mutation was probably from the mitochondria gene or intron of the chromosome. Next, we conducted whole gene sequencing to find the mutation of patient III-2. It is well-known that RP is characterized by the growing loss of photoreceptors and RPE, indicating a significant genetic and phenotypic heterogeneity (4). Till now, more than 70 genes have been implicated in this disorder from published studies (18). Several genes relevant to the tri-snRNP complex of the spliceosome have been demonstrated for adRP (19). Moreover, the age of onset, progress, and the eventual blindness of these patients varies according to the different genotypes. Therefore, the detection and definition of the genotypes of these patients are essential to provide prophylactic advice and available genetic therapy in the near future. Under the optimization of next-generation sequencing technology in recent years, WES analysis has been applied for determining mutation in exons and splicing sites at the genomic scale (20).

The knocking down of endogenous SNRNP200 in zebrafish using MO has been previously demonstrated to cause retinal degeneration, indicating that an insufficient gene quantity of SNRNP200 could result in RP in patients (17). The dominant-negative effect is probably the pathogenic mechanism associated with SNRNP200^c.C6088T^ as indicated by the zebrafish study. The phenotype of the zebrafish model injected with SNRNP200^c.C6088T^ mutant mRNA showing high ratios of deformation and loss of photoreceptors identified by immunofluorescence were recorded. They were not noticed in those injected with SNRNP200^WT^ mRNA, implying that the mutation functions as an antimorph or a neomorph. Previous research showed that the zebrafish model injected with a sub-lethal dosage of the SNRNP200-MO and the retina-specific phenotype were affected with reduced or even loss of a generation of photoreceptor, which resembles the manifestation resulting from zebrafish overexpressing SNRNP200^c.C6088T^ mRNA. These results indicate that SNRNP200^c.C6088T^ acts as an antimorph rather than a neomorph, being expected to produce distinct phenotypes compared with a morphant model. Certainly, the co-injection of SNRNP200^c.C6088T^ and MO overwhelmingly increased the death ratio of the morphants in our study. Given that the morphant zebrafish is not a real null allele for SNRNP200, this observation is presumably caused by the dominant-negative effect of SNRNP200^c.C6088T^ on the sustained function of the orthologous zebrafish SNRNP200 gene, which shows 89% identity with human SNRNP200.

The mechanism of the mutations in pre-mRNA splicing genes with universal transcription in almost every cell causing retina-specific phenotypes are speculated to be via these particular mechanisms. Chen et al. (21) reported that the retina displays one of the highest rates of oxidative metabolism of all cells in the body with the consecutive renewal of OS that highly depends on the pre-mRNA splicing of many genes. Hence, the systemic defects resulting from such splicing mutations could only be compromising to the retina as supported by some data (22, 23). Moreover, the retina might be highly sensitive to the fidelity mechanisms of splicing which is influenced by the mutation of RP genes (21). As is reported, the gene SNRNP200 is essential for the proofreading of pre-mRNA splicing (24–26). More importantly, our experiment findings indicate that SNRNP200 expresses extensively in the CMZ area where retinal stem cells (RSCs) and retinal progenitor cells (RPCs) are located, which precisely coordinate proliferation and differentiation of cells in the retina of zebrafish. Therefore, we conclude that the SNRNP200^c.C6088T^ is likely to impair the development of the retina, including the photoreceptor by the influence of RSCs and RPCs. Subsequently, we are going to investigate how the SNRNP200^c.C6088T^ influence these cell developments by generating the zebrafish SNRNP200 mutant model.

In summary, SNRNP200^c.C6088T^, a novel RP-associated pre-mRNA splicing gene-locus caused the typical clinical sign of RP. Considering the genetic findings and the phenotypes of the corresponding zebrafish models, the mutation of SNRNP200 locus identified in this study, like mutations of PRPF4, is evidenced to cause RP via a dominant-negative effect. In the future, more scanning of the SNRNP200 locus in RP patients should be performed to find its function in the etiology.

## Conclusion

In conclusion, this study reports a novel heterozygous SNRNP200^c.C6088T^ mutation causing the typical adRP in a Chinese family. We found that this mutation could significantly cause the deformation of zebrash larvae with the overexpression of SNRNP200 p.Arg2030Cys mRNA and increase the death rate of zebrash when the endogenous SNRNP200 p.Arg2030Cys was blocked. All these findings indicate that this mutation causes RP via a dominant-negative effect.

## Data Availability Statement

The datasets presented in this study can be found in online repository, it can be found using the accession number BioProject ID: PRJNA686229.

## Ethics Statement

The studies involving human participants were reviewed and approved by First Affiliated Hospital of Shantou University Medical College (SUMC) Institutional Review Board. The patients/participants provided their written informed consent to participate in this study. The animal study was reviewed and approved by First Affiliated Hospital of Shantou University Medical College (SUMC) Institutional Review Board. Written informed consent was obtained from the owners for the participation of their animals in this study.

## Author Contributions

TZ and ZL: conceptualization, data curation, formal analysis, investigation, methodology, and writing—original draft. JB: conceptualization, project administration, resources, and supervision. XZha: investigation and methodology. XZhe: investigation, methodology, and validation. NL: investigation, methodology, software, and visualization. LL and YC: conceptualization, funding acquisition, project administration, resources, supervision, and writing—review and editing. All authors contributed to the article and approved the submitted version.

## Conflict of Interest

ZL was employed by company Berry Genomics Co., Ltd. The remaining authors declare that the research was conducted in the absence of any commercial or financial relationships that could be construed as a potential conflict of interest.

## Nomenclature

RP, retinitis pigmentosa

pre-mRNA, pre-message RNA

SNRNP200, small nuclear ribonucleoprotein 200 kDa

WES, Whole exome sequencing

MO, Morpholino oligonucleotide

VFs, visual fields

RPE, retinal pigment epithelium

adRP, autosomal dominant RP

snRNPs, small nuclear ribonucleoprotein particles

OCT, optical coherence tomography

Q-PCR, Quantitative real-time PCR

OS/IS, outer/inner segments.
